# Intraoperative nociception and postoperative inflammation associated with the suppression of major complications due to thoracic epidural block after pleurectomy/decortication for malignant pleural mesothelioma under general anesthesia: A retrospective observational study

**DOI:** 10.1097/MD.0000000000034832

**Published:** 2023-09-01

**Authors:** Yuka Hamanaka, Wakana Ueda, Kanako Taki, Ken Onoe, Yuka Matsuki, Hiroai Okutani, Ryusuke Ueki, Munetaka Hirose

**Affiliations:** a Department of Anesthesiology and Pain Medicine, Hyogo Medical University Faculty of Medicine, Nishinomiya, Hyogo, Japan; b Department of Anesthesiology & Reanimatology, Faculty of Medicine Sciences, University of Fukui, Eiheiji-cho, Fukui, Japan.

**Keywords:** epidural anesthesia, inflammation, nociception, postoperative complications, regional anesthesia, White blood cell count

## Abstract

A recent study showed that thoracic epidural block (TEB) suppressed the occurrence of major complications after pleurectomy/decortication (P/D) for malignant pleural mesothelioma (MPM) under general anesthesia. To investigate the mechanisms underlying the correlation, both acute inflammatory status and intraoperative nociception were evaluated in the present study. In a single-institutional observational study, consecutive adult patients undergoing P/D were enrolled from March 2019 to April 2022. Perioperative acute inflammatory status was evaluated using differential White blood cell (WBC) counts and serum concentration of C-reactive protein (CRP) both before and after the surgery on postoperative day (POD) 1. The averaged value of nociceptive response index during surgery (mean NR) was obtained to evaluate the level of intraoperative nociception. Multivariable logistic regression analysis was performed to determine the association between perioperative variables and major complications Postoperative major postoperative complication was defined as Clavien-Dindo grades ≥ III. We conducted this study with 97 patients. After logistic regression analysis showed that general anesthesia without TEB was a sole risk factor for major complications, patients were divided into 2 groups: general anesthesia with and without TEB. The incidence of major complications was significantly lower in patients with TEB (33.3%, n = 33) than in those without TEB (64.1%, n = 64, *P* < .01). Although there was no significant difference in the CRP level between 2 groups, the lymphocyte-to-monocyte ratio (LMR) on POD 1 in patients with TEB was significantly higher than that in patients without TEB (*P* = .04). The mean NR was significantly lower in patients with TEB than that in those without TEB (*P* = .02). Both lower mean NR during surgery and higher LMR on POD 1 are likely associated the suppression of major complications due to TEB after P/D under general anesthesia. Decreases in the postoperative acute inflammatory response, caused by the reduction of intraoperative nociception due to TEB, may help suppress major complications after P/D.

## 1. Introduction

Pleurectomy/decortication (P/D) is performed as a surgical treatment for malignant pleural mesothelioma (MPM), consisting of en bloc resection of the lung where the visceral and parietal pleura are removed together with the pericardium and/or diaphragm, if required.^[1–3]^ The incidence of major complications within 30 days after surgery is significantly high in patients undergoing P/D (35.9%–51.5%).^[2,3]^ A recent study demonstrated that the incidence of major complications after P/D was 32.3% in patients with thoracic epidural block (TEB), which was significantly lower than 63.1% in patients without TEB under general anesthesia.^[4]^ The underlying mechanisms of associations between TEB and the inhibitory rate of postoperative major complications, however, have not been revealed.

To reveal factors related to mechanisms underlying the suppression of major complications due to TEB, we conducted this retrospective observational study in patients undergoing P/D for MPM with curative intent.

## 2. Methods

### 2.1. Ethics

This single-institutional observational study was approved by the Ethics Committee of Hyogo Medical University (Ethical Committee number 3138) on March 4, 2019. The requirement for written informed consent for study participation was waived by the institutional ethics committee.

### 2.2. Patients

We enrolled consecutive patients who received P/D from March 2019 to April 2022. Since Hyogo Medical University Hospital is one of high-volume centers well experienced in P/D in Japan,^[3]^ we performed this study as a single-institutional study. Exclusion criteria were age <19 years.

### 2.3. Data collection

The primary outcome of this study was to reveal factors related to the association between TEB and the suppression of postoperative major complications. To assess this, we obtained perioperative data of age, sex, body mass index (BMI), American Society of Anesthesiologists—physical status (ASA-PS), emergency status, surgical procedure, anesthetic management, serum concentrations of C-reactive protein (CRP) before surgery and on postoperative day (POD) 1, and complications occurring within 30 days after surgery from our institutional medical records. To evaluate the level of intraoperative nociception, the averaged values of nociceptive response (NR) index from the start to the end of surgery.^[5]^ To seek factors related to the association between TEB and postoperative major complications, we additionally obtained differential White blood cell (WBC) counts, neutrophil-to-lymphocyte ratio, and lymphocyte-to-monocyte ratio (LMR) before and after surgery on postoperative day (POD) 1 to evaluate perioperative acute inflammatory status. These data were retrospectively obtained from the patients medical records.

### 2.4. Postoperative complications

Postoperative complications were graded according to the Clavien-Dindo classification, which includes 7 grades: grade I, any deviation from the normal postoperative course; grade II, alteration of the normal postoperative course; grade III, complications that require interventions under local anesthesia (IIIa) or general/epidural anesthesia (IIIb); grade IV, life-threatening complications with single-organ (IVa) or multi-organ dysfunction (IVb); and grade V, death within 30 days after surgery^[6]^ for complications were defined as Clavien-Dindo grade IIIa or higher.

### 2.5. Anesthetic management during surgery

None of the patients received premedication. General anesthesia was induced with propofol along with fentanyl and rocuronium. Total intravenous anesthesia was maintained with continuous infusions of propofol and remifentanil, in addition to fentanyl and rocuronium during surgery. Doses of remifentanil and fentanyl were adjusted to maintain mean blood pressure within the range of ± 20% of the pre-anesthesia level. Bispectral index (BIS) was maintained between 40 and 60 by adjusting the target dose of propofol. Red blood cells (RBC) were transfused as required to maintain hemoglobin concentrations above 7–8 g·dL^−1^.

Additional TEB was performed if deemed appropriate by the attending anesthesiologists due to patient co-morbidities or his/her experience. A thoracic epidural catheter was inserted under infiltration anesthesia using 1% mepivacaine before induction of general anesthesia. Continuous infusions of 4 mL·hr^−1^ of 0.15% levobupivacaine, along with fentanyl at 20 μg·hr^−1^, in addition to bolus injection of 1% mepivacaine were administered during general anesthesia in patients receiving TEB.

Postoperatively, all patients received multimodal analgesia. Patients who received TEB continued to receive continuous epidural infusion of levobupivacaine and fentanyl at the same doses, with patient-controlled bolus injection of 3 mL, until POD3. On the other hand, in patients, who did not receive TEB, a continuous intravenous infusion of fentanyl at 25 to 50 μg·hr^−1^ was administered until POD3 for postoperative analgesia. In all patients, additional analgesia, if the patient required it, was achieved using intravenous infusion of acetaminophen, oral administration of loxoprofen or tramadol, or transdermal fentanyl.

### 2.6. NR index values during surgery

The NR index, as a dimensionless number between 0 and 1, is a quantitative value for assessing physiologic responses to the balance between nociception secondary to surgical stimuli and anti-nociception due to anesthesia during surgery under general anesthesia.^[7,8]^ The NR index was calculated every 1 minute during surgery using the NR formula, which includes the intraoperative hemodynamic variables of heart rate (HR), systolic blood pressure (SBP), and perfusion index (PI). Increase in nociception or decrease in anti-nociception increases the NR value. SBP was measured directly using a catheter in the radial artery contralateral to the side of surgery. PI values were derived from the plethysmographic pulse wave amplitude via a pulse oximetry probe attached to a finger on the side contralateral to the surgery. The equation for NR was installed on the anesthesia information managing system (ORSYS; Philips Japan, Tokyo, Japan), and NR index values were stored in our medical records every 1 minute. The averaged values of NR (mean NR) from the start of surgery to the end of surgery in each patient were also obtained using Vi-Pros data-search software (Dowell Co. Ltd., Sapporo, Japan).

### 2.7. Sample size calculation

Sample size was calculated using PS Power and Sample Size Calculations version 3.0 software (Dupont WD and Plummer WD, Vanderbilt University Medical Center, Nashville, TN). Calculations were performed assuming a type I error probability of 0.05 and power of 0.8. A previous study reported that the incidence of postoperative complications, defined as Clavien-Dindo grade ≥ III, was 32.3 % in patients with TEB and 63.1% in patients without TEB.^[4]^ We thus assumed that the probabilities of postoperative complications were 30% and 60% in patients with or without TEB in the present study. Required sample size was then estimated to be 42 participants in each group and 84 in total. Factoring in a 5% dropout rate, we aimed to collect data from at least 89 participants in this study.

### 2.8. Statistical analysis

Statistical testing was performed using JMS Pro version 14.2.0 (SAS Institute Inc. Cary, NC). Multivariable logistic regression analysis was used to determine the association between regional anesthesia and postoperative major complications. To exclude confounding effects between regional anesthesia and preoperative and intraoperative variables, we selected preoperative variables of age, sex, BMI, ASA-PS and preoperative CRP levels, and intraoperative variables of mean NR, and RBC transfusion volume, as candidate variables for multivariable logistic regression analysis. If multicollinearity was present between these variables based on a variance inflation factor of <10, variables were not included in the analysis.^[9]^ The results of multivariable logistic regression analysis are presented as odds ratios with 95% confidence intervals (CIs). Comparisons of 2 variables were performed using Mann–Whitney *U* test or χ^2^ test as appropriate, where *P* values < .05 were considered significant.

## 3. Results

This study was performed following to the Strengthening the Reporting of Observational Studies in Epidemiology (STROBE) guideline. We enrolled 122 patients undergoing P/D from March 2019 to April 2022. However, 3 patients were missing data for serum CRP concentrations on POD1 and NR index, and 22 patients were missing data for perioperative differential WBC counts. Thereafter, we performed the present study in 97 patients.

Table 1 shows patients’ characteristics and perioperative variables. All surgeries were electively performed in patients with ASA-PS II or III. The number of patients who received TEB was 33 (34.0%), and those who did not receive it was 64 (66.0%). The incidence of major complications within 30 days after these surgeries, defined as Clavien-Dindo grade ≥ III, was occurred in 57 patients (53.6% [95% CI 43.7%–63.2%]). Major postoperative complications included air leak (n = 45), respiratory failure (n = 5), reoperation (n = 2), brain infarction (n = 2), cardiac failure (n = 1), and others (n = 2).

**Table 1:** Patients’ characteristics and perioperative variables.

Preoperative variables	
Age, yr	68 (7)
Male/Female, n (%)	77/20 (79.4/ 20.6)
BMI, kg·m^−2^	22.8 (2.7)
ASA-PS II/ III, n (%)	56/ 41 (57.8/ 42.3)
Serum concentration of CRP, mg·dL^−1^	1.08 (1.76)
WBC count, 10^2^·μL^−1^	62.2 (18.6)
Neutrophil count, 10^2^·μL^−1^	41.5 (17.1)
Lymphocyte count, 10^2^·μL^−1^	14.0 (5.2)
Monocyte count, 10^2^·μL^−1^	4.9 (1.7)
NLR	3.6 (3.3)
LMR	3.1 (1.3)
**Intraoperative variables**	
With/ without TEB, n (%)	33/ 64 (34.0/ 66.0)
Mean NR	0.853 (0.021)
Continuous dose of remifentanil, μg·kg^−1^·min^−1^	0.168 (0.057)
Total amount of fentanyl, μg·kg^−1^	9.3 (3.8)
Total amount of rocuronium, mg·kg^−1^	3.4 (0.8)
Duration of surgery, min	454 (98)
Duration of anesthesia, min	568 (102)
Blood loss, mL	1853 (1172)
RBC transfusion volume, mL	949 (546)
Urine volume, mL	707 (459)
**Postoperative variables**	
Serum concentration of CRP on POD1, mg·dL^−1^	5.50 (2.00)
WBC count on POD 1, 10^2^·μL^−1^	116.1 (32.2)
Neutrophil count on POD 1, 10^2^·μL^−1^	102.6 (30.4)
Lymphocyte count on POD 1, 10^2^·μL^−1^	7.8 (3.1)
Monocyte count on POD 1, 10^2^·μL^−1^	5.7 (2.2)
NLR on POD 1	15.1 (7.2)
LMR on POD 1	1.6 (0.8)
Clavien-Dindo grade < III/ ≥ III, n (%)	45/ 57 (46.4/ 53.6)

Data are expressed as numbers (%) or means (SD).

ASA-PS = American Society of Anesthesiologists—Physical Status, CRP = C-reactive protein, LMR = lymphocyte-to-monocyte ratio, NLR = neutrophil-to-lymphocyte ratio, NR = Nociceptive Response, POD = postoperative day, RBC = red blood cells, SD = standard deviation, TEB = thoracic epidural block, WBC = White blood cell.

### 3.1. Logistic regression analysis for associations between TEG and major complications

We performed multivariable logistic regression analyses to determine the association between regional anesthesia and major complications. Eight variables of age ≥ 70 years, male sex, BMI ≥ 25 kg·m^−2^, ASA-PS ≥ III, preoperative CRP levels ≥ 1.00 mg·dL^−1,[10]^ regional anesthesia,^[11]^ mean NR ≥ 0.85^[12]^ and RBC transfusion volume ≥ 1200 mL^[13]^ were selected as candidate variables. There was no multicollinearity existed between these candidate variables. Multivariable logistic regression revealed that absence of epidural block was an independent risk factor for major complications (Table 2).

**Table 2:** Multivariable logistic regression analysis of pre- and intraoperative variables related to major complications.

Pre- and intraoperative variables	Odds ratio [95% CI]	*P*
Age ≥ 75, yr	1.22 [0.38–3.91]	.73
Male sex	1.49 [0.49–4.58]	.48
BMI ≥ 25, kg·m^−2^	0.51 [0.18–1.45]	.21
ASA-PS ≥ III	0.96 [0.37–2.44]	092
Preoperative CRP level ≥ 1.00, mg·dL^−1^	1.52 [0.58–3.99]	.39
TEB	0.23 [0.09–0.60]	<.01*
Mean NR ≥ 0.85	0.47 [0.19–1.16]	.10
RBC transfusion volume ≥ 1200, mL	1.28 [0.45–3.70]	.64

ASA-PS = American Society of Anesthesiologists—Physical Status, BMI = body mass index, CI = confidence interval, CRP = C-reactive protein, NR = nociceptive response, RBC = red blood cells, TEB = thoracic epidural block.

Statistical significance was defined at

* *P* < .01.

### 3.2. Comparisons of perioperative variables between patients with and without TEG

Next, we divided all patients into 2 groups with or without TEB (n = 33 and n = 64, respectively) (Table 3). Although there were no significant differences in preoperative variables between 2 groups, the mean NR index during surgery, continuous dose of remifentanil, and total amount of fentanyl during anesthesia were significantly lower in patients with TEB than in those without TEG. Among postoperative variables, LMR on POD 1 was significantly higher in patients with TEG than in those without TEG. The incidence of major complications after surgery was 35.3% (95% CI 21.5%–52.1%) in patients with TEG, which was significantly lower than 63.5% (95% CI 51.1%–74.3%) in those without TEG.

**Table 3:** Comparisons of perioperative variables between patients with and without epidural block.

Perioperative variables	Without TEB, n = 64	With TEB, n = 33	*P*
**Preoperative variables**			
Age, yr	68 (8)	67 (6)	.48
Male/Female, n (%)	50/14 (78.1/ 21.9)	27/6 (81.8/ 18.2)	.67
BMI, kg·m^−2^	22.9 (2.7)	22.6 (2.8)	.61
ASA-PS II/ III, n (%)	33/31 (51.6/ 48.4)	23/ 10 (69.7/ 32.4)	.09
Serum concentration of CRP, mg·dL^−1^	0.96 (1.63)	1.33 (2.00)	.19
WBC count (SD) in 10^2^·μL^−1^	61.7 (20.5)	63.1 (14.6)	.36
Neutrophil count, 10^2^·μL^−1^	41.3 (19.3)	41.9 (12.0)	.47
Lymphocyte count, 10^2^·μL^−1^	13.5 (5.4)	15.0 (4.6)	.06
Monocyte count, 10^2^·μL^−1^	4.9 (1.9)	4.8 (1.2)	.79
NLR	3.8 (3.9)	3.1 (1.8)	.44
LMR	2.9 (1.2)	3.3 (1.3)	.13
**Intraoperative variables**			
Mean NR	0.856 (0.022)	0.846 (0.017)	.02*
Continuous dose of remifentanil, μg·kg^−1^·min^−1^	0.191 (0.049)	0.121 (0.043)	<.01**
Total amount of fentanyl, μg·kg^−1^	10.6 (3.2)	6.6 (3.5)	<.01**
Total amount of rocuronium, mg·kg^−1^	3.4 (0.9)	3.2 (0.6)	.42
Duration of surgery, min	459 (101)	446 (92)	.77
Duration of anesthesia, min	563 (104)	579 (98)	.38
Blood loss, mL	1847 (1064)	1866 (1376)	.77
RBC transfusion volume, mL	916 (560)	1035 (510)	.24
Urine volume, mL	715 (487)	692 (406)	.95
**Postoperative variables**			
Serum concentration of CRP on POD1, mg·dL^−1^	5.57 (2.12)	5.35 (1.78)	.48
WBC count on POD 1, 10^2^·μL^−1^	117.8 (33.6)	112.9 (29.5)	.56
Neutrophil count on POD 1, 10^2^·μL^−1^	104.1 (31.3)	99.6 (28.9)	.53
Lymphocyte count on POD 1, 10^2^·μL^−1^	7.6 (3.1)	8.2 (3.1)	.46
Monocyte count on POD 1, 10^2^·μL^−1^	5.9 (2.4)	5.1 (1.6)	.15
NLR on POD 1	15.8 (7.8)	13.7 (5.9)	.23
LMR on POD 1	1.5 (0.8)	1.7 (0.7)	.04*
Clavien-Dindo grade < III/≥ III, n (%)	23/ 41 (35.9/64.1)	22/11 (66.7 33.3)	<.01**

Data are expressed as numbers (%) or means (SD).

ASA-PS = American Society of Anesthesiologists—Physical Status, BIS = bispectral index, BMI = body mass index, CRP = C-reactive protein, LMR = lymphocyte-to-monocyte ratio, NLR = neutrophil-to-lymphocyte ratio, NR = Nociceptive Response, POD = postoperative day, RBC = red blood cells, SD = standard deviation, TEB = thoracic epidural block, WBC = White blood cell. Comparisons of 2 variables were performed using Mann–Whitney *U* test or χ^2^ test as appropriate.

Significant differences were considered at

* *P* < .05 and

** *P* < .01.

## 4. Discussion

TEB reduced mean NR during surgery while simultaneously increasing the LMR on POD 1, subsequent decrease in the incidence of major complications after P/D was observed in the present study. The significant decrease in mean NR in patients with TEB represents lower levels of nociception during surgery than that in patients without TEB.^[5]^ On the other hand, the increase in the LMR indicates an increase in lymphocyte counts or a decrease in monocyte, correlating with anti-inflammation, which suppresses morbidity or mortality.^[14–16]^ Previous studies have revealed that increases in sympathetic activity, accompanied by excessive nociception, contribute to hematopoiesis in the bone marrow, including neutrophils and monocytes,^[17,18]^ This excessive acute inflammation has been shown to cause postoperative complications.^[5]^ Therefore, the suppression of nociception caused by TEB, which was observed as lower values of mean NR in the present study, is likely associated with a reduction in acute inflammation. This anti-inflammation helps suppress postoperative major complications.

In the present study, there was no significant difference in the serum concentration of CRP after surgery between patients with and without TEB. The serum concentration of CRP serves as a marker of inflammation, and acute phase responses to surgical trauma lead to the production of CRP by the liver, resulting in increased CRP level.^[19]^ Since the P/D for MPM, which exhibited the mean NR of approximately 0.85 in the present study, is considered one of the highest-risk surgical procedures in terms of postoperative morbidity and mortality,^[20]^ it is possible that despite the suppression of nociception in patients with TEB, the significantly high surgical invasiveness during P/D might elevate CRP levels to a similar extent as observed in patients without TEB.

A limitation of this study is that it is a retrospective observational study based on perioperative data. The study includes patients who underwent P/D from January 2022 to April 2022, as well as those included in our previous study from March 2019 to December 2021.^[4]^ While the duration of patient enrollment in the present study was longer than in the previous study,^[4]^ the number of analyzed patients in the present study was smaller due to a large volume of missing data on differential WBC counts. Further investigations are needed to determine the precise mechanisms by which TEB exerts suppressive effects on major complications after P/D in a prospective randomized controlled study in the future.

## 5. Conclusion

Both lower mean NR during surgery and higher LMR on POD 1 are likely correlated with the suppression of postoperative major complications caused by TEB in patients undergoing P/D for MPM under general anesthesia. Decreases in the postoperative acute inflammatory response, caused by the reduction of intraoperative nociception due to TEB, may help suppress major complications after P/D.

## Author contributions

**Conceptualization:** Munetaka Hirose.

**Data curation:** Yuka Hamanaka, Wakana Ueda, Kanako Taki, Ken Onoe, Yuka Matsuki, Munetaka Hirose.

**Formal analysis:** Munetaka Hirose.

**Methodology:** Ken Onoe, Munetaka Hirose.

**Supervision:** Hiroai Okutani, Ryusuke Ueki.

**Validation:** Hiroai Okutani, Ryusuke Ueki.

**Writing – original draft:** Yuka Hamanaka, Wakana Ueda, Kanako Taki.

**Writing – review & editing:** Hiroai Okutani, Ryusuke Ueki, Munetaka Hirose.
